# Advanced paternal age and risk of cancer in offspring

**DOI:** 10.18632/aging.202333

**Published:** 2020-12-19

**Authors:** Yangyang Sun, Xu Li, Wei Jiang, Yuanming Fan, Qiong Ouyang, Wei Shao, Raphael N. Alolga, Yuqiu Ge, Gaoxiang Ma

**Affiliations:** 1 State Key Laboratory of Natural Medicines, School of Traditional Chinese Pharmacy, China Pharmaceutical University, Nanjing, China; 2 Department of Urology, The Third Affiliated Hospital of Soochow University, Changzhou, China; 3 Department of Science and Technology, Sir Run Run Hospital, Nanjing Medical University, Nanjing, China; 4 Department of Public Health and Preventive Medicine, Wuxi School of Medicine, Jiangnan University, Wuxi, China

**Keywords:** paternal age, cancer risk, offspring, cohort study

## Abstract

Many risk factors of cancer have been established, but the contribution of paternal age in this regard remains largely unexplored. To further understand the etiology of cancer, we investigated the relationship between paternal age and cancer incidence using PLCO cohort. Cox proportional hazards models were performed to assess the association between paternal age and the risk of cancers. During follow-up time (median 11.5 years), 18,753 primary cancers occurred. Paternal age was associated with reduced risk of cancers of the female genitalia (HR, 0.79; 95%CI, 0.66-0.94; *P* = 0.008) as well as cancers of the respiratory and intrathoracic organs (HR, 0.78; 95%CI, 0.63-0.97; *P* = 0.026). The association was stronger for lung cancer (HR, 0.67; 95%CI, 0.52-0.86; *P* = 0.002). The subgroup analysis suggested that age, gender, smoking and BMI were related to the decreased cancer incidence of the respiratory and intrathoracic organs, lung and the female genitalia. Positive linear associations were observed between paternal age and cancer incidence of the female genitalia, respiratory and intrathoracic organs and the lungs. These findings indicate that advanced paternal age is an independent protective factor against various cancers in offspring.

## INTRODUCTION

Per the report of the International Agency for Research on Cancer (IARC), cancer remains one of the leading causes of death, with an estimated 18.1 million new cases and 9.6 million deaths worldwide for the year 2018 [1]. It is estimated that about 1.81 million new cancer cases and 0.61 million cancer deaths are projected to occur in the United States in 2020 [2]. With knowledge of the established risk factors of cancer such as smoking, obesity and diabetes, life style, genetic susceptibilities, family history and so on [3–6], the etiology of cancer continues to be explored.

The impact of age of mother at pregnancy on the health of offspring is well documented. However, not much is known about the influence of paternal age on the health of the offspring. Several studies have investigated the role of paternal age on the incidence of disease in offspring [7–11]. It is well known that advanced paternal age is associated with some disorders in the offspring including schizophrenia [12], intellectual disability [13] and achondroplasia [14]. Notably, the outcome of a cohort study in Sweden demonstrated that advanced paternal age is an important independent risk factor for schizophrenia [15]. Besides, moderate significant relations have been noted in childhood cancers, but no firm conclusions could be made for other types of cancer [16, 17]. Numerous studies have established the link between the risk of childhood malignancy and paternal age [16, 18, 19]. Recently, the results of a case-control study showed a positive correlation between paternal age (as a risk factor) and breast cancer [20]. However, a critical evaluation of the design of this study and its small sample size bring their results into question.

The present study examined the association between paternal age and risk of cancers in adult offspring in a large sample of middle-aged and old subjects who participated in the Prostate, Lung, Colorectal and Ovarian (PLCO) cancer screening trial.

## RESULTS

### Baseline characteristics of study population

**Figure 1:**
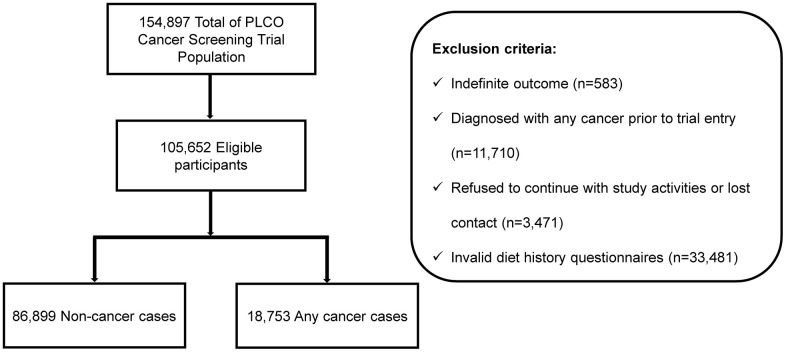
**Flowchart of inclusion/exclusion criteria.** Eligible participants from the PLCO cohort were selected by inclusion criteria.

The inclusion/exclusion criteria are summarized in Figure 1. After selection, our study included 105,652 participants (mean age at baseline, 62.5 years; 53,473 [50.6%] women; 96,230 [91.1%] non-Hispanic white). The mean duration from randomization to completing the SQ was 9.1 years, with a median follow-up time of 11.5 years. In the study cohort, 18,753 individuals were diagnosed with cancer during follow-up, including 846 individuals who had cancer of the female genitalia (502 individuals with corpus uteri cancer [59.3%], 243 individuals with ovarian cancer [28.7%] and other sites [12%]) and 1,873 individuals had cancers of the respiratory and intrathoracic organs (120 individuals with larynx cancer [6.4%], 1,666 individuals with bronchus and lung cancers [88.9%] and other sites [4.6%]). Demographic characteristics of the study population are summarized in Table 1. There were significant differences in age, sex, BMI, race, education, marital status, smoking status, alcohol consumption, family history of any cancer and total fruit intake between cancer cases and cancer-free participants.

**Table 1:** Characteristics of the PLCO cohort.

**Characteristics**	**No. of participants (%)**	**No. of cancers (%)**	***P***
Age			**<0.001**
55-59 years	35,721 (33.8)	4,900 (26.1)	
60-64 years	32,981 (31.2)	5,966 (31.8)	
65-69 years	23,774 (22.5)	5,045 (26.9)	
70+ years	13,176 (12.5)	2,842 (15.2)	
Sex			**<0.001**
Male	52,179 (49.4)	11,495 (61.3)	
Female	53,473 (50.6)	7,258 (38.7)	
BMI			**<0.001**
<18.5 kg/m^2^	665 (0.6)	94 (0.5)	
18.5-25 kg/m^2^	34,325 (32.9)	5,821 (31.5)	
25-30 kg/m^2^	44,714 (42.9)	8,286 (44.8)	
30+ kg/m^2^	24,576 (23.6)	4,295 (23.2)	
Race			**<0.001**
White, Non-Hispanic	96,230 (91.1)	17,301 (92.3)	
Black, Non-Hispanic	3,459 (3.3)	612 (3.3)	
Hispanic	1,530 (1.4)	229 (1.2)	
Asian	3,701 (3.5)	508 (2.7)	
Pacific Islander	478 (0.5)	71 (0.4)	
American Indian	216 (0.2)	30 (0.2)	
Education			**<0.001**
≤11years	6,380 (6.1)	1,252 (6.7)	
Completed high school	24,448 (23.2)	4,128 (22.1)	
Post high school	13,667 (13.0)	2,442 (13.0)	
College	60,954 (57.8)	10,892 (58.2)	
Tobacco smoking status			**<0.001**
Never smokers	50,153 (47.5)	7,784 (41.5)	
Current smokers	9,811 (9.3)	2,314 (12.3)	
Former smokers	45,674 (43.2)	8,651 (46.1)	
Marital status			**<0.001**
Married or living as married	82,924 (78.6)	15,023 (80.3)	
Widowed	8,454 (8)	1,414 (7.6)	
Divorced	9,957 (9.4)	1,580 (8.4)	
Separated	803 (0.8)	126 (0.7)	
Never married	3,321 (3.1)	569 (3.0)	
Alcohol drinking intensity (g/day)			**<0.001**
Never	28,808 (27.3)	4,898 (26.1)	
0-5	41,147 (38.9)	7,024 (37.5)	
5-10	10,333 (9.8)	1,845 (9.8)	
10-20	10,098 (9.6)	1,841 (9.8)	
20-30	7,790 (7.4)	1,517 (8.1)	
30+	7,476 (7.1)	1,628 (8.7)	
Family history of cancer			**<0.001**
No	46,217 (43.9)	7,809 (41.7)	
Yes	59,144 (56.1)	10,901 (58.3)	
Total fruit intake (g/day), Median (IQR)	231.80 (129.09, 359.88)	228.37 (126.95, 357.13)	**0.010**
Total vegetable intake (g/day), Median (IQR)	242.93 (159.21, 359.49)	242.81 (159, 357.96)	0.656

### Paternal age and systemic cancer incidence

The results of cox proportional hazards regression model for paternal age and the risk of cancers of organs or system in offspring are presented in Table 2. Our results show that paternal age is significantly associated with reduced risk of cancers of the female genitalia (HR, 0.79; 95%CI, 0.66-0.94; *P* = 0.008). For cancers of the respiratory and intrathoracic organs, paternal age was associated with a lower hazard rate for every 10 years of paternal age (HR, 0.78; 95%CI, 0.63-0.97; *P* = 0.026). There were no significant associations between paternal age and the risk of other systemic cancers in all crude and adjusted regression models.

**Table 2:** Paternal age and the cancer risk of organs or system in PLCO cohort.

**Primary sites**	**cases/cohort**	**HR^1^**	**95%CI^1^**	***P*^1^**	**HR^2^**	**95%CI^2^**	***P*^2^**
Any cancers^a^	18,753/105,652	0.98	0.95-1.01	0.215	0.98	0.94-1.01	0.162
Lip, oral cavity and pharynx^b^	195/105,652	1.03	0.74-1.44	0.865	1.11	0.79-1.56	0.562
Digestive organs^c^	2,372/105,652	1.02	0.92-1.12	0.751	1.05	0.94-1.17	0.386
Respiratory and intrathoracic organs^d^	1,873/105,652	0.96	0.84-1.09	0.538	0.78	0.63-0.97	**0.026**
Hematopoietic and reticuloendothelial systems^e^	851/105,652	0.98	0.83-1.15	0.815	0.98	0.83-1.15	0.785
Skin^f^	955/105,652	0.95	0.83-1.08	0.432	0.94	0.82-1.08	0.388
Connective subcutaneous and other soft tissues^g^	76/105,652	0.70	0.43-1.14	0.153	0.68	0.42-1.12	0.128
Female genitalia^h^	846/53,473	0.89	0.76-1.04	0.142	0.79	0.66-0.94	**0.008**
Male genital organs^i^	5,959/52,179	0.98	0.93-1.03	0.419	1.00	0.89-1.11	0.945
Urinary tract^j^	1,387/105,652	0.98	0.87-1.11	0.743	0.96	0.85-1.08	0.505
Eye, brain and other parts of central nervous system^k^	190/105,652	1.00	0.66-1.53	0.985	1.03	0.68-1.58	0.876
Thyroid and other endocrine glands^l^	172/105,652	0.79	0.56-1.14	0.210	0.79	0.55-1.14	0.204
Lymph nodes^m^	596/105,652	1.08	0.90-1.30	0.398	1.09	0.90-1.32	0.368

Abbreviations: HR, hazard ratio; CI, confidence interval.

Model 1: adjusted for maternal age, age, sex, race/ethnicity.

Model 2 of a: model 1 plus BMI, randomization arm, cigarette smoking status, alcohol drinking intensity, family history of cancer, total vegetable and fruit intake, processed red meat intake, nonalcohol total energy, grain intake, physical activity 1+time per month.

Model 2 of b & c: model 2 of a plus grain intake, using aspirin, weight lifting and aerobics.

Model 2 of d: model 1 plus BMI, randomization arm, cigarette smoking status, alcohol drinking intensity, family history of cancer, total vegetable and fruit intake, nonalcohol total energy, beta-carotene intake, physical activity 1+time per month, cigarettes smoked per day, pack-year cigarette smoking, age started smoking, age stopped smoking.

Model 2 of e: model 1 plus BMI, randomization arm, cigarette smoking status, alcohol drinking intensity, family history of cancer, total vegetable and fruit intake, nonalcohol total energy.

Model 2 of f: model 1 plus BMI, randomization arm, education, cigarette smoking status, alcohol drinking intensity, family history of cancer total vegetable and fruit intake.

Model 2 of h: model1 plus BMI, randomization arm, cigarette smoking status, alcohol drinking intensity, family history of cancer, total vegetable and fruit intake, history of diabetes, removed ovaries, having a hysterectomy, age at menopause, pregnancies, taking female hormones, family history of endometrial cancer, using aspirin, the cross-product term of maternal age and age.

Model 2 of i: model 1 plus BMI, randomization arm, cigarette smoking status, education, family history of prostate cancer, history of diabetes, frequency of strenuous activity, beta-carotene intake, processed red meat intake, lactose intake, vitamin D intake, total fat intake, poly-unsaturated fatty acids from plant sources intake, having any prostate surgeries, age at vasectomy, having enlarged prostate or benign prostatic hypertrophy.

Model 2 of g, j, k and l: same as model 2 of f.

Model 2 of m: model 1 plus BMI, randomization arm, cigarette smoking status, alcohol drinking intensity, family History of lymphoma cancer, total vegetable and fruit intake.

### Paternal age and site-specific cancer incidence

We further examined the relationship between paternal age and the specific location of cancer based on anatomical coding. As shown in Table 3, a similar association of advanced paternal age and the risk of lung cancer was observed (HR, 0.67; 95%CI, 0.52-0.86; *P* = 0.002). Since the lungs form part of the respiratory and intrathoracic organs, we used the respiratory and intrathoracic organs regression model to adjust the covariates in the lung regression model. We also examined the relationship between paternal age and the risk of cancer in specific sites of other systems or organs. However, no significant trends were observed in other specific sites.

**Table 3:** Paternal age and the cancer risk of sites in PLCO cohort.

**Primary sites**	**cases/cohort**	**HR^1^**	**95%CI^1^**	***P*^1^**	**HR^2^**	**95%CI^2^**	***P*^2^**
Esophagus^a^	144/105,652	1.06	0.66-1.69	0.808	1.14	0.69-1.89	0.614
Stomach^b^	206/105,652	0.93	0.64-1.36	0.715	0.93	0.61-1.40	0.716
Small intestine^c^	69/105,652	1.26	0.78-2.01	0.344	1.31	0.80-2.14	0.277
Colon^d^	1,092/105,652	0.96	0.84-1.11	0.593	1.00	0.86-1.16	0.992
Rectosigmoid junction^e^	64/105,652	1.34	0.84-2.12	0.218	1.44	0.90-2.32	0.130
Rectum^f^	238/105,652	0.89	0.65-1.21	0.458	0.90	0.65-1.24	0.519
Liver^g^	77/105,652	0.66	0.29-1.50	0.319	0.81	0.34-1.93	0.635
Pancreas^h^	362/105,652	1.38	1.04-1.83	0.023	1.34	0.98-1.84	0.069
Larynx^i^	120/105,652	0.90	0.58-1.42	0.657	1.09	0.60-2.00	0.770
Bronchus^j^	97/105,652	1.09	0.54-2.17	0.817	0.61	0.15-2.53	0.500
Lung^k^	1,569/105,652	0.94	0.82-1.09	0.414	0.67	0.52-0.86	**0.002**
Bone marrow^l^	798/105,652	0.99	0.84-1.17	0.892	0.98	0.83-1.16	0.846
Female breast^m^	3,053/53,473	1.00	0.93-1.08	0.981	0.99	0.91-1.08	0.836
Corpus uteri^n^	502/53,473	0.91	0.75-1.10	0.335	0.82	0.66-1.02	0.068
Ovary^o^	243/53,473	0.89	0.65-1.22	0.480	0.72	0.50-1.05	0.089
Prostate^p^	5,930/52,179	0.98	0.92-1.03	0.391	1.00	0.89-1.12	0.960
Kidney^q^	433/105,652	1.03	0.83-1.28	0.755	1.00	0.80-1.25	0.989
Bladder^r^	890/105,652	0.98	0.85-1.14	0.831	0.98	0.84-1.15	0.840
Brain^s^	150/105,652	0.99	0.59-1.68	0.973	1.03	0.61-1.76	0.904
Thyroid^t^	151/105,652	0.82	0.57-1.18	0.279	0.80	0.55-1.15	0.229

Model 1 is adjusted for maternal age, age, sex, race/ethnicity.

Model 2 of a, b, c, d, e, f, g and h: same as model 2 of digestive organs in Table 2.

Model 2 of i and j: model 2 of respiratory and intrathoracic organs in Table 2.

Model 2 of k: same as model 2 of digestive organs in Table 2 except family history of lung cancer.

Model 2 of l: same as model 2 of hematopoietic and reticuloendothelial systems in Table 2.

Model 2 of m: same as model 2 of female genitalia in Table 2 except family history of breast cancer.

Model 2 of n: same as model 2 of female genitalia in Table 2.

Model 2 of o: same as model 2 of female genitalia in Table 2 except family history of ovarian cancer.

Model 2 of p: same as model 2 of male genital organs in Table 2.

Model 2 of q: same as model 2 of urinary tract in Table 2.

Model 2 of r: model 1 plus BMI, randomization arm, cigarette smoking status, alcohol drinking intensity, family history of bladder cancer, total vegetable and fruit intake.

Model 2 of s: same as model 2 of eye, brain and other parts of central nervous system in Table 2.

Model 2 of t: same as model 2 of thyroid and other endocrine glands in Table 2.

### Subgroup analysis

**Figure 2:**
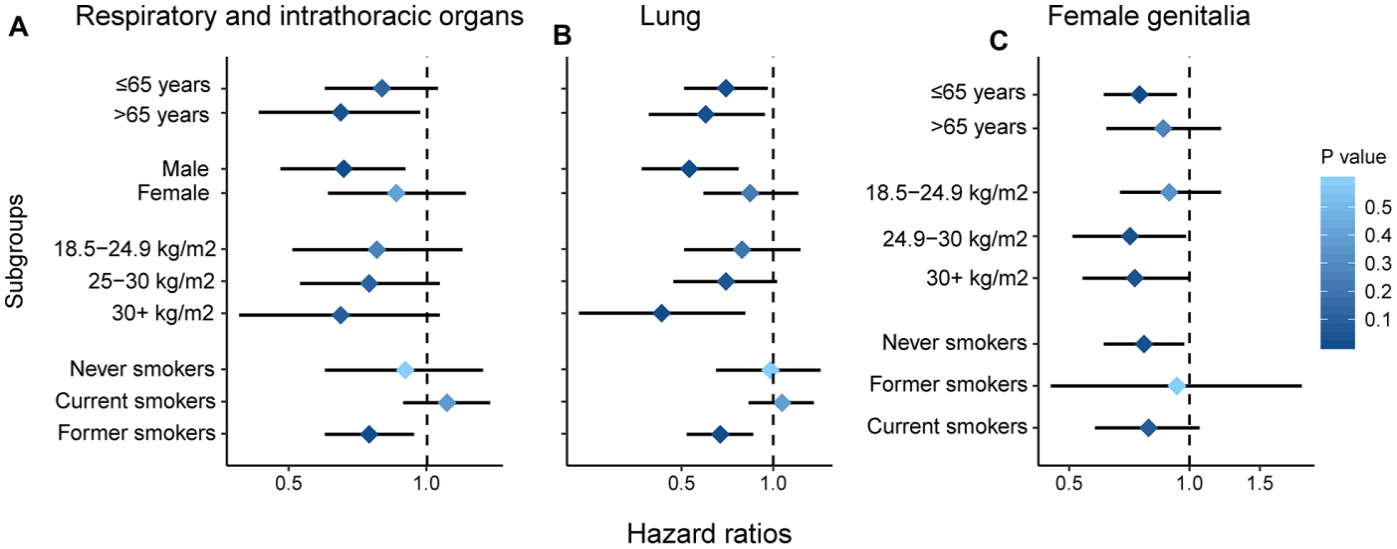
**Subgroup analysis by age, gender, smoking and BMI.** Respiratory and intrathoracic organs (**A**); lung (**B**); female genitalia (**C**).

Additionally, we performed stratified analyses to evaluate the impact of paternal age on the risk of cancers of the respiratory and intrathoracic organs, female genitalia and lungs (Table 4 and Figure 2). For the incidence of cancers of the respiratory and intrathoracic organs, paternal age was associated with reduced risk among individuals of age > 65 years (HR, 0.65; 95%CI, 0.43-0.97; *P* = 0.033), in male (HR, 0.66; 95%CI, 0.48-0.90; *P* = 0.009) and former smokers (HR, 0.75; 95%CI, 0.60-0.94; *P* = 0.012). For cancers of the female genitalia, reduced risk was noted among individuals of age > 65 years (HR, 0.75; 95%CI, 0.61-0.93; *P* = 0.008), with 25 > BMI > 30 kg/m^2^ (HR, 0.71; 95%CI, 0.51-0.98; *P* = 0.037) and never smokers (HR, 0.77; 95%CI, 0.61-0.97; *P* = 0.029). For lung cancer, it was associated with reduced risk among individuals of age > 65 years (HR, 0.70; 95%CI, 0.51-0.96; *P* = 0.026) and age > 65 years (HR, 0.60; 95%CI, 0.39-0.94; *P* = 0.024), in male (HR, 0.53; 95%CI, 0.37-0.77; *P* = 0.001), with BMI > 30 kg/m^2^ (HR, 0.43; 95%CI, 0.23-0.81; *P* = 0.008) and former smokers (HR, 0.67; 95%CI, 0.52-0.86; *P* = 0.002).

**Table 4:** Subgroup analysis by age, gender, BMI and smoking status.

**Subgroup**	**Respiratory and intrathoracic organs**			***P***	**Lung**			***P***	**Female genitalia**			***P***
	**cases/cohort**	**HR**	**95%CI**		**cases/cohort**	**HR**	**95%CI**		**cases/cohort**	**HR**	**95%CI**	
Age												
≤65 years	1,108/63,228	0.80	0.60-1.06	0.117	910/63,030	0.70	0.51-0.96	**0.026**	590/33,551	0.75	0.61-0.93	**0.008**
>65 years	765/25,544	0.65	0.43-0.97	**0.033**	659/25,438	0.60	0.39-0.94	**0.024**	256/13,510	0.86	0.62-1.20	0.384
Sex												
Male	1,140/41,824	0.66	0.48-0.90	**0.009**	904/41,588	0.53	0.37-0.77	**0.001**	-	-	-	-
Female	733/46,948	0.86	0.61-1.22	0.397	665/46,880	0.84	0.59-1.21	0.356	846/47,061	-	-	-
BMI^a^												
≤18.5 kg/m^2^	9/580	^b^	^b^	^b^	9/580	^b^	^b^	^b^	9/469	^b^	^b^	^b^
18.5-24.9 kg/m^2^	672/28,556	0.78	0.51-1.20	0.260	581/28,465	0.79	0.51-1.23	0.304	295/18,130	0.89	0.67-1.20	0.465
25-30 kg/m^2^	814/37,862	0.75	0.53-1.07	0.110	677/37,725	0.70	0.47-1.03	0.068	256/16,686	0.71	0.51-0.98	**0.037**
>30 kg/m^2^	354/20,635	0.65	0.39-1.07	0.092	284/20,565	0.43	0.23-0.81	**0.008**	282/11,225	0.53	0.54-1.00	0.051
Smoking status												
Never smokers	197/42,566	0.90	0.60-1.33	0.592	146/42,515	0.98	0.65-1.43	0.932	484/27,326	0.77	0.61-0.97	**0.029**
Current smokers	727/8,224	1.11	0.89-1.38	0.368	614/8,111	1.07	0.83-1.36	0.602	59/3,729	0.93	0.45-1.91	0.843
Former smokers	949/37,023	0.75	0.60-0.94	**0.012**	809/37,832	0.67	0.52-0.86	**0.002**	303/15,702	0.79	0.58-1.06	0.121

^a^ Calculated as weight in kilograms divided by height in meters squared.

^b^ Insufficient cases.

Respiratory and intrathoracic organs model: same as model 2 of respiratory and intrathoracic organs in Table 2.

Lung model: same as model 2 of lung in Table 2.

Female genitalia model: same as model 2 of female genitalia in Table 2.

### Dose-response analysis

**Figure 3:**
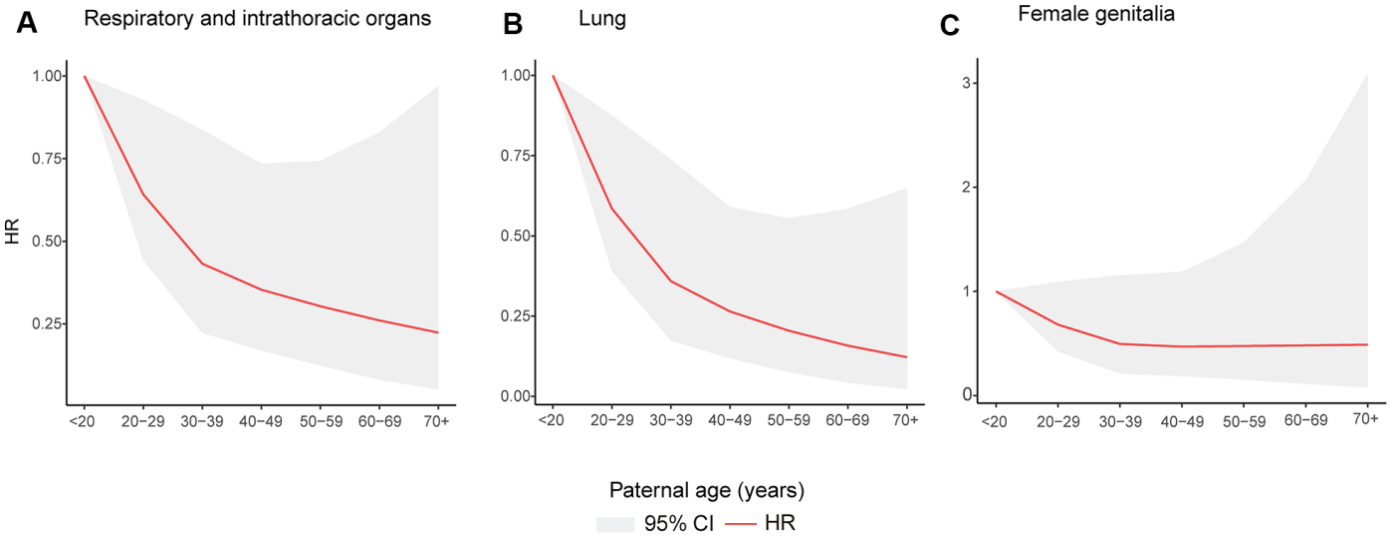
**Restricted cubic spline models for association between paternal age and incident of cancer of as follows.** Cancers of the respiratory and intrathoracic organs (**A**); lung cancer (**B**); cancers of the female genitalia (**C**).

The cubic spline models showed a positive linear relationship between paternal age and the risk of cancers of the respiratory and intrathoracic organs (*P* for non-linearity= 0.330), lung cancer (*P* for non-linearity= 0.410) and cancers of the female genitalia (*P* for non-linearity= 0.313) (Figure 3). When we treated paternal age as a continuous variable, every 10 years of increase in paternal age was associated with a 22%, 33% and 21% decrease in cancer risk of the respiratory and intrathoracic organs, lung and female genitalia, respectively (data not shown in figure).

## DISCUSSION

In this PLCO cohort study, we found that higher paternal age was associated with lower risk of cancers of the female genitalia in offspring. Higher paternal age was also associated with lower risk of cancers of the respiratory and intrathoracic organs (including lung cancer). Additionally, our subgroup analysis also suggested that higher paternal age had a strong inverse association with the risk of above-mentioned cancers, and this may be associated with age, sex, BMI and smoking status. Dose-response analysis also confirmed that paternal age was an independent protective factor against cancers of the respiratory and intrathoracic organs, lung cancer and cancers of the female genitalia.

The effect of paternal age on the prevention of different cancers is controversial. A case-control study in Korea revealed that advanced paternal age increased the risk of breast cancer in their female offspring, but this adverse association was not observed in our study [21]. Additionally, a cohort study of 1,000,000 men found that advanced paternal age was linearly associated with lower risk of testicular germ cell tumors [22]. A similar association was observed in a nationwide register-based cohort study, which revealed that higher paternal age was associated with lower risk of the central nervous system neoplasms [16]. A Danish population-based registry study found that advanced paternal age was associated with reduced risk of childhood Wilms’ tumor [23]. Although previous studies have reported paternal age as a protective factor against cancer, similar reports have been rare, suggesting that the protective role of paternal age in cancer might not be well understood and requires further investigation.

Our findings may be related to the genetics of human telomere length (TL). Previous studies reported that the dual properties of short telomeres and inhibited telomerase may make humans and large, long-lived mammals relatively resistant to cancer [24]. Telomerase activity in mammals was found to be negatively correlated with body size [25, 26], while TL was negatively correlated with lifetime [27]. Thus, inhibiting telomerase and short telomeres limits the human ability to replicate. In this way, short TL may inhibit the accumulation of new mutations and reduce the likelihood of oncogenic transformation in large, long-lived mammals [24]. However, environmental factors such as socioeconomic status, smoking, sedentary lifestyle, energy intake, and perhaps mental stress are also associated with short telomere length [28–34]. Some studies have suggested that TL at birth is the primary determinant of TL in adults [35, 36]. Hence, TL was considered a highly heritable trait [37]. A LTL (leukocyte telomere length) Genome Wide Association Study showed that the same cluster of LTL-associated alleles is a risk factor for lung cancer, when the joint effect of the alleles results in a comparatively short LTL, thereby reducing the risk of cancer [38]. A pooled analysis of three prospective cohorts and a large case-control study identified the significant association between telomere length and risk of lung cancer [39, 40]. The results of our subgroup analysis revealed that the association between telomere length and risk of lung cancer was significant in male. This may be explained by the incidence of long telomere length in women than men due the prevalence of estrogen in the former [41]. Modern humans have short telomeres and exhibit inhibited telomerase activity in somatic tissues. The combination of short telomeres and inhibited telomerase activity may make humans relatively resistant to cancer [42–44]. We therefore speculate that paternal age as a protective factor could be due to the presence of short telomeres.

This study presents with certain strengths that are worth mentioning. First of all, through a prospective study design, data were collected from pre-diagnosis questionnaires. Therefore, we can exclude the correlative effect of recall bias. We looked at all the cancers and specific cancer sites. The sample size of the cohort and the number of cancer patients are relatively large. To our knowledge, this study is the first prospective follow-up study to evaluate the relationship between paternal age and cancer risk in adults.

Our study also has several limitations. Firstly, when obtaining self-reported data, it is important to be aware of the limitations of the data, such as under-reporting of variables of interest, the potential impact of this error in the analysis, and the potential bias of some missing values. For example, environmental exposure factors for fathers were missing from the PLCO data, making it impossible to correct for biases caused by potential confounders and to examine whether there is an interaction with paternal age. Some specific family cancer history variables were also missing. Secondly, we found that higher paternal age was associated with lower HRs of cancers of the female genitals in offspring, but this association was not observed for any specific cancers of the female reproductive organ. Interestingly, we found corpus uteri model was close to the significance result (*P* = 0.068). The reason is that there are a few cases of specific cancers of the female genitalia (e.g., ovaries) in this cohort, and we hope to use multicenter, large-sample data to further verify our findings. Also, it cannot be ignored that when information was collated during the PLCO cohort study, paternal age was registered as a categorical variable. For this reason, we could not examine it as a continuous variable in the model, which might result in loss of some information. Thirdly, the relatively low incidence of lung cancer in this study may yield biased results (Supplementary Table 4). Previous studies have shown that the PLCO cohort was a controlled trial to determine whether or not certain screening examinations reduced mortality due to prostate, lung, colorectal and ovarian cancers [21, 22]. Participants of the intervention arm of the study underwent a chest x-ray at baseline and did same annually for 2 years. In particular, participants classified as "smokers" underwent an additional chest x-ray at year 3 for lung screening. Lung screening interventions reduce the incidence of lung cancer and influence participants' smoking behavior. This therefore could explain the low percentage of active smokers in the cohort, leading to a lower incidence of lung cancer. Finally, the study population was elderly, which suggests that there may be residual confounding of socio-economic factors. Nevertheless, causality specifically attributable to paternal age is not proven by this work, and deserves further consideration.

## CONCLUSIONS

In this cohort study, we found a significant association of advanced paternal age with reduced risk of cancers of the female genitalia, cancers of the respiratory and intrathoracic organs (including lung cancer) among individuals of 55 years and above in the PLCO Cancer Screening Trial.

## MATERIALS AND METHODS

### Study design

The PLCO cancer screening trial is a randomized trial designed to assess the effect of screening methods on the mortality of prostate, lung, colorectal and ovarian cancers [45, 46]. The trial began in November 8, 1993, with about 155,000 men and women aged between 55 and 74 from 10 screening centers in the United States. The initial trial was approved by Institutional Review Boards at the National Cancer Institute at all research centers.

### Evaluation of demographic and lifestyle variables

The baseline questionnaire (BQ) was given to all participants upon enrollment and provided the demographic information of participants, including age, gender, group, race/ethnicity, marital status, educational status, personal and family medical history, tobacco smoking status, medication use, anthropometry and other selected life style factors. About 97% of the participants returned their questionnaires.

### Assessment of paternal age and dietary variables

The variable of paternal age was derived from supplemental questionnaire (SQ), which was introduced in 2006, 13 years after enrollment and overlapped with information collected in the BQ. The SQ added some variables, which were not collected in the BQ (i.e., physical activity, history of diabetes and family history of endometrial cancer). Paternal age was classified as less than 20, from 20 to 29, 30 to 39, 40 to 49, 50 to 59, 60 to 69, more than 70 years. The diet history questionnaire (DHQ) included analysis-ready dietary variables. DHQ was offered to both arms of the trial starting in 1998 and 77% of all subjects of the trial completed the DHQ.

### Outcome assessment and study population

In the PLCO trial, reports of cancers collected were not limited to annual study update questionnaire, but also from relatives, friends, or physicians and death certificates from National Death Indices. All reports of cancers and any medical records were extracted. The follow-up lasted until the recording of diagnostic information was completed, which included the type of cancer, date of diagnosis, hospital or clinical diagnosis and physician contact information. Cutoff of follow-up was ascertained until the occurrence of one of the following events: diagnosis of cancers, death from any cause, or the end of follow-up as determined by availability of data ready for analysis, whichever came first. All cancer sites had International Classification of Disease for Oncology, third edition (ICD-O-3) codes based on the initial medical records. The anatomical codes of the organ or system of tumors are shown in Supplementary Table 1. The anatomical codes for the specific sites of the tumors are shown in Supplementary Table 2. A total of 154,897 participants took part in the trial, as described previously [45, 46]. Individuals with uncertain diagnostic outcome were excluded (n = 583). Individuals who had cancer before enrollment (n = 11,710), refused to continue with study activities or lost contact and did not complete baseline questionnaire (n = 3,471), or did not have valid DHQ (n = 33,481) were excluded. A total of 105,652 participants were qualified for further analysis.

### Statistical analysis

The median (interquartile range) was used to describe the consumption of fruits and vegetables. Mann-Whitney U test for continuous variables was used to examine the differences between cancer patients and cancer-free controls. Categorical variables (demographic, anthropometric, and lifestyle characteristics) of the study subjects were compared by χ^2^ test. Cox proportional hazards regression analysis was performed to calculate hazard ratios (HRs) and the corresponding 95% confidence intervals (CIs) to assess the relationship between paternal age and risk of cancer.

The stepwise regression approach was used to pre-determine potential confounding factors, which was based on previous literature and availability of data. The crude models (Model 1) of all cancers included adjustment for age, sex, race, and maternal age. To estimate the potential heterogeneity of different cancers, we adjusted for different confounding factors in another model (Model 2). The covariates of all models are shown in Supplementary Table 3.

The crude models of specific anatomical sites of cancers included adjustment for maternal age, age, sex, and race. Multivariate models of specific anatomic cancers had the same adjustment variables as the models of corresponding organ or system. The main difference between the models was that, for each type of cancer, family history was duly taken into consideration and adjusted for. For instance, the lung cancer model adjusted for a family history of lung cancer, while the breast cancer model adjusted for a family history of breast cancer. The same was done for ovarian cancer and cancer of the bladder.

To quantify dose-response relationships, we used restricted cubic spline models with four knots at the 5^th^, 35^th^, 65^th^, and 95^th^ centiles to examine the associations between paternal age and incidence of respiratory and intrathoracic organs, as well as lung and female genitalia cancers after full adjustment. Less than 20 years was treated as the reference for all restricted cubic spline analyses.

All statistical tests were two-sided, and *P* < 0.05 was considered statistically significant. Analyses were performed using R software (version 3.6.1).

## Supplementary Material

Supplementary Tables
