# Supplementary material for: Advanced paternal age and risk of cancer in offspring
Source: Aging (Albany NY). 2020 Dec 19;13(3):3712–25. doi: 10.18632/aging.202333 (PMC7906132; doi:10.18632/aging.202333)
Supplement: Supplementary Tables [file aging-13-202333-s001.pdf]

SUPPLEMENTARY TABLES

Supplementary Table 1. Anatomical codes of the organ or system of tumors.

Organs or systems	ICD-O-3 codes
Lip, oral cavity and pharynx	C00.0 to C14.8
Digestive organs	C15.0 to C26.9
Respiratory and intrathoracic organs	C30.0 to C39.9
Hematopoietic and reticuloendothelial system	C42.0 to C42.4
Skin system	C44.0 to C44.9
Connective subcutaneous and other soft tissues	C49.0 to C49.9
Breast organ	C50.0 to C50.9
Female genital organs	C51.0 to C58.9
Male genital organs	C60.0 to C63.9
Urinary tract	C64.9 to C68.9
Eye, brain and other parts of central nervous system	C69.0 to C72.9
Thyroid and other endocrine glands	C73.9 to C75.9
Lymph nodes	C77.0 to C77.9

Supplementary Table 2. Anatomical codes of the specific sites of the tumors.

Specific-sites	ICD-O-3 codes
Esophagus	C15.0 to C15.9
Stomach	C16.0 to C16.9
Small intestine	C17.0 to C17.9
Colon	C18.0 to C18.9
Rectosigmoid junction	C19.9
Rectum	C20.9
Liver	C22.0
Pancreas	C25.0 to C25.9
Larynx	C32.0 to C32.9
Bronchus	C34.0
Lung	C34.1 to C34.9
Bone marrow	C42.1
Corpus uteri	C54.0 to C54.9
Ovary	C56.9
Prostate	C61.9
Kidney	C64.9
Bladder	C67.0 to C67.9
Brain	C71.0 to C71.9
Thyroid	C73.9

Supplementary Table 3. Covariates for models of all systems or organs.

System or organs	Covariates
Any cancers	Maternal age, age, sex, race, BMI, randomization arm, cigarette smoking status, alcohol drinking intensity, family history of cancer, total vegetable and fruit intake, processed red meat intake, nonalcohol total energy, grain intake, physical activity 1+time per month
Lip, oral cavity and pharynx	Maternal age, age, sex, race, BMI, randomization arm, cigarette smoking status, alcohol drinking intensity, family history of cancer, total vegetable and fruit intake, processed red meat intake, nonalcohol total energy, grain intake, using aspirin, physical activity 1+time per month, weight lifting and aerobics.
Digestive organs	Same as Lip, oral cavity and pharynx model.
Respiratory and intrathoracic organs	Maternal age, age, sex, race, BMI, randomization arm, cigarette smoking status, alcohol drinking intensity, family history of cancer, total vegetable and fruit intake, nonalcohol total energy, beta-carotene intake, physical activity 1+time per month, cigarettes smoked per day, pack-year cigarette smoking, age started smoking, age stopped smoking.
Hematopoietic and reticuloendothelial systems	Maternal age, age, sex, race, BMI, randomization arm, cigarette smoking status, alcohol drinking intensity, family history of cancer total vegetable and fruit intake, nonalcohol total energy
Skin	Maternal age, age, sex, race, BMI, randomization arm, cigarette smoking status, alcohol drinking intensity, family history of cancer total vegetable and fruit intake, education
Connective subcutaneous and other soft tissues	age, sex, race, BMI, randomization arm, cigarette smoking status, alcohol drinking intensity, family history of cancer total vegetable and fruit intake
Breast	Maternal age, age, race, BMI, randomization arm, cigarette smoking status, alcohol drinking intensity, family history of breast cancer, total vegetable and fruit intake, total fiber intake, history of diabetes, taking birth control pills, age at birth of first child, number of children born, removed ovaries.
Female genital organs	Maternal age, age, race, BMI, randomization arm, cigarette smoking status, alcohol drinking intensity, family history of cancer, total vegetable and fruit intake, history of diabetes, removed ovaries, having a hysterectomy, age at menopause, pregnancies, taking female hormones, family history of endometrial cancer, using aspirin.
Male genital organs	Maternal age, age, race, BMI, randomization arm, cigarette smoking status, education, family history of prostate cancer, history of diabetes, frequency of strenuous activity, beta-carotene intake, processed red meat intake, lactose intake, vitamin D intake, total fat intake, poly-unsaturated fatty acids from plant sources intake, having any prostate surgeries, age at vasectomy, having enlarged prostate or benign prostatic hypertrophy.
Urinary tract	Same as connective subcutaneous and other soft tissues model
Eye, brain and other parts of central nervous system	Same as connective subcutaneous and other soft tissues model
Thyroid and other endocrine glands	Same as connective subcutaneous and other soft tissues model
Lymph nodes	Same as connective subcutaneous and other soft tissues model plus family history of lymphoma

Supplementary Table 4. Distribution of the number of lung cancers by paternal age.

Paternal age (years)	Cases	Participants
<20	1	1,001
20-29	285	33,603
30-39	243	30,416
40-49	83	10,022
50-59	11	1,506
60-69	3	219
70+	3	180
